# Supplementary material for: Generalist species drive microbial dispersion and evolution
Source: Nat Commun. 2017 Oct 27;8:1162. doi: 10.1038/s41467-017-01265-1 (PMC5660117; doi:10.1038/s41467-017-01265-1)
Supplement: Supplementary file 1 — Supplementary Information [file 41467_2017_1265_MOESM1_ESM.pdf]

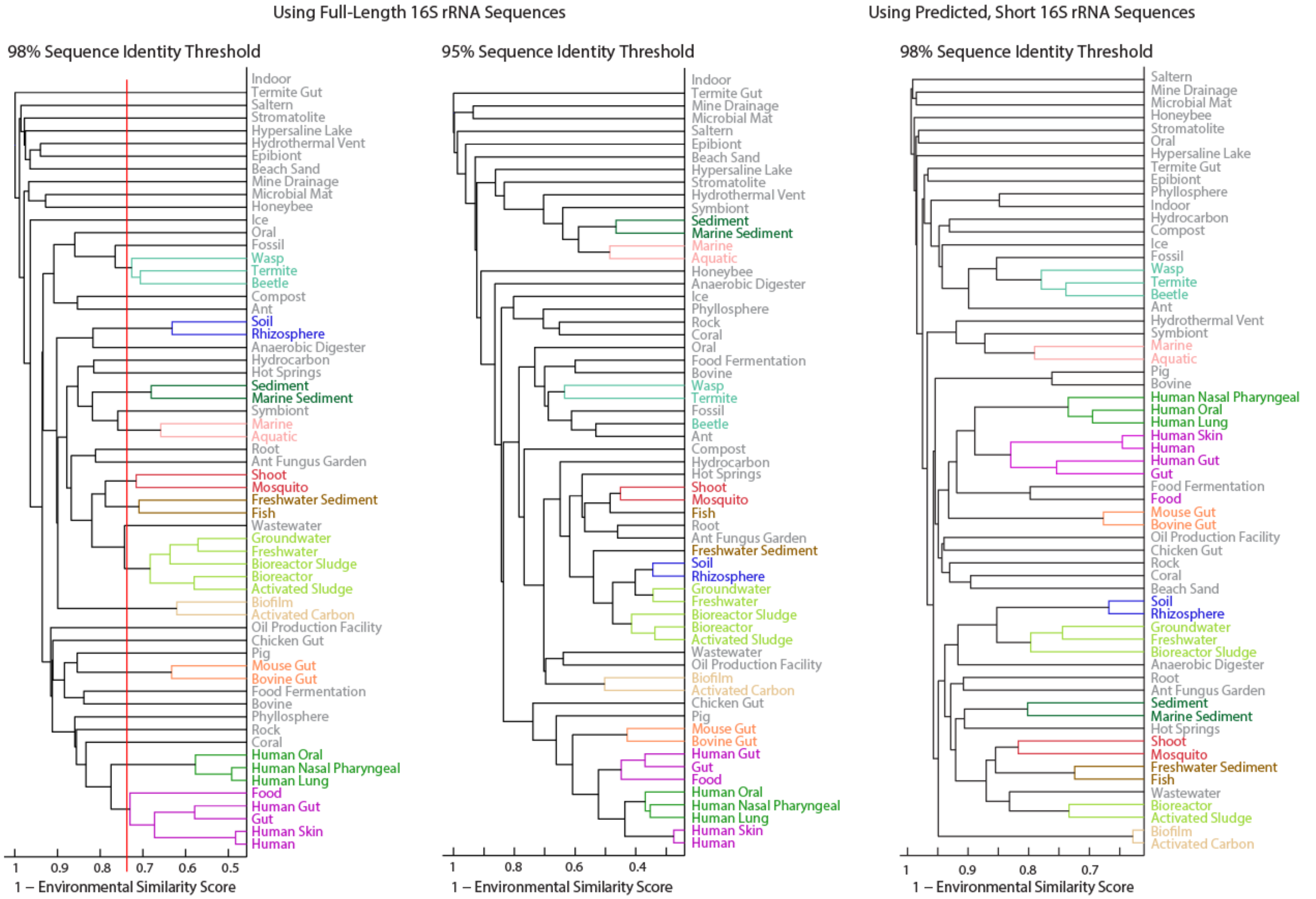

Supplementary Figure 1 | Clustering of environments with 'environmental similarity score'. For the leftmost and middle panels, full-length 16S rRNA sequences from SILVA database were used and the sequence identity thresholds for calculating similarity score were set at 98% and 95%, respectively. For the rightmost panel, the sequence identity threshold was set at 98% but predicted 16S rRNA fragments were used instead of full-length sequences. The color scheme for displaying environment names is the same as that in Figure 1. Environments belonging to singleton clusters are shown in grey.

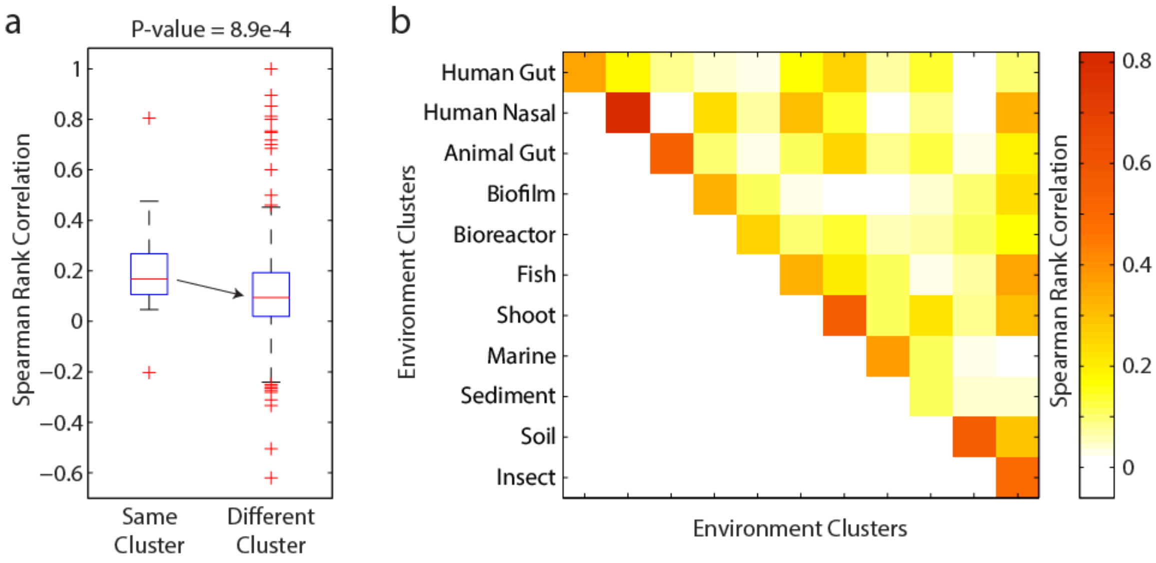

Supplementary Figure 2 | Comparison of species abundance profiles between environments. (**a**) Boxplot comparing the Spearman rank correlation between the species abundance profiles of two environments within the same environment cluster, 'Same Cluster', or from different environment clusters, 'Different Cluster'. Mann-Whitney U test p-value is indicated. The blue boxes and red bars indicate the 25th-75th percentile region and the median, respectively. The black whiskers cover roughly 99.3% of the distribution. (**b**) Heat map showing average Spearman rank correlation between the species abundance profiles of two environments within the same environment cluster (diagonal entries), or between different environment clusters.

a

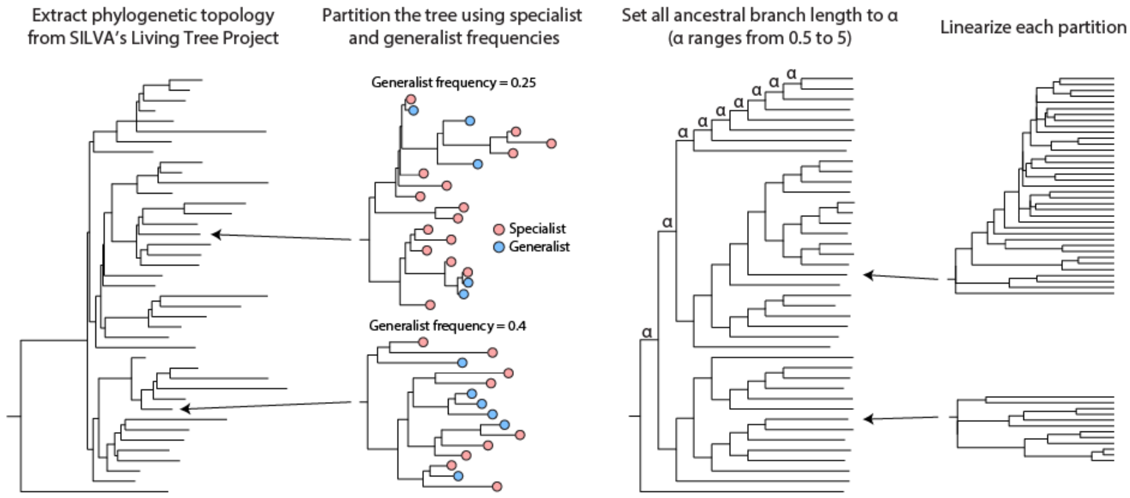

b

Using all species (statistics derived from a 1,000-step Markov Chain Monte Carlo simulation)

α	λ_s	μ_s	λ_g	μ_g	$t_{s\to g}$	$t_{g\to s}$
0.2	3.34±1.63	13.16±5.71	38.53±3.50	27.67±6.50	3.51±1.80	11.17±2.67
0.5	1.91±1.21	20.36±3.63	35.74±2.69	22.55±4.41	2.50±1.72	14.98±1.73
1.0	3.44±2.03	15.33±3.03	39.16±1.15	30.58±1.87	4.18±1.09	11.78±2.06
2.5	2.50±1.04	17.04±1.89	37.51±0.52	27.56±0.48	4.20±0.89	12.90±0.70
5.0	1.20±0.95	24.44±2.11	34.57±0.15	18.03±0.28	1.92±0.77	17.95±0.83

Using 80% of all species (statistics derived from 100 subsamples)

α	λ_s	μ_s	λ_g	μ_g	$t_{s\to g}$	$t_{g\to s}$
0.5	1.75±1.58	15.74±3.49	33.85±1.91	24.08±4.72	4.19±1.08	12.71±2.40
5.0	1.88±2.00	16.24±6.34	33.13±3.79	23.05±9.03	4.20±2.51	13.21±3.92

Supplementary Figure 3 | Phylogenetic reconstruction and evolutionary characteristics estimation under different parameters. (**a**) Divide-and-conquer scheme for linearizing the archaeal-bacterial phylogenetic tree. After extracting the phylogenetic topology from SILVA's Living Tree Project, the tree was partitioned using frequencies of specialist and generalist species (Methods). Individual partitions were then linearized separately and subsequently joined together using the original topology and fixed ancestral branch length, α. (**b**) Estimations of evolutionary characteristics were performed at various values of ancestral branch length, α. Stability and robustness of the estimated rates were assessed with 1,000-step Markov Chain Monte Carlo simulations and 100 subsamples at 80% sampling rate. Average values and standard deviations are shown here. λ_s and λ_g are speciation rates for specialist and generalist states, respectively. μ_s and μ_g are extinction rates. *t*'s are state transition rates.

Using all species (statistics derived from a 1,000-step Markov Chain Monte Carlo simulation)

α	λ_s	μ_s	λ_g	μ_g	$t_{s\to g}$	$t_{g\to s}$
0.5	3.00±0.72	18.37±1.58	33.86±2.13	6.56±3.46	4.49±0.76	35.14±2.35
5.0	3.90±0.24	11.78±0.18	43.79±0.19	29.25±0.51	5.78±0.22	25.21±0.96

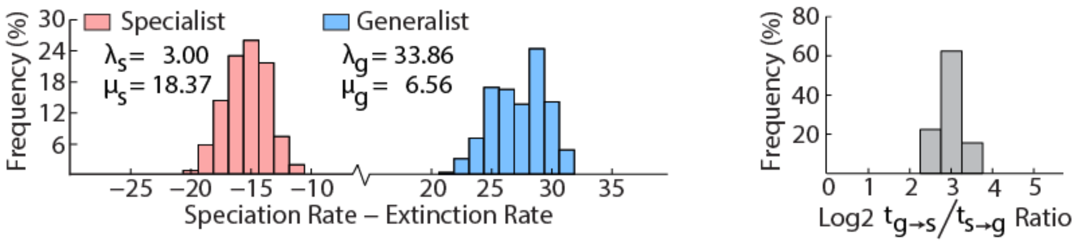

Supplementary Figure 4 | Re-estimation of evolutionary characteristics using a 95% sequence identity threshold for mapping 16S rRNA sequences. This is the same as in Figure 3 but performed at a 95% sequence identity threshold instead of 98%. Bar plots consist of data obtained at $\alpha = 0.5$. The same conclusions of i) higher speciation rates for generalists, ii) positive expansion rate for generalists, iii) negative expansion rates for specialists, and iv) enrichments of generalist-to-specialist transformation rates over the reverse transitions were observed.

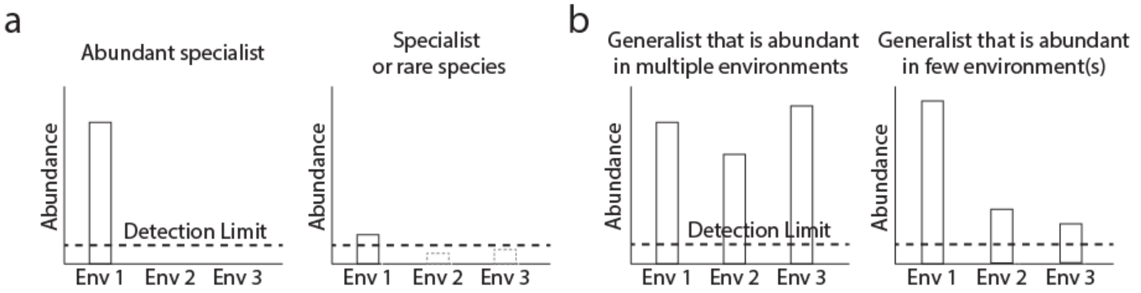

c

Removed 5% of specialists with lowest abundance and 5% of generalists with low entropy
Statistics derived from a 1,000-step Markov Chain Monte Carlo simulation

α	λ_s	μ_s	λ_g	μ_g	$t_{s\to g}$	$t_{g\to s}$
0.5	2.05±1.13	19.17±3.19	35.24±2.24	22.70±3.67	3.09±1.54	14.82±1.59
5.0	2.57±0.99	12.77±1.69	39.94±0.12	32.91±0.31	5.15±0.61	10.66±0.42

Removed 10% of specialists with lowest abundance and 10% of generalists with low entropy
Statistics derived from a 1,000-step Markov Chain Monte Carlo simulation

α	λ_s	μ_s	λ_g	μ_g	$t_{s\to g}$	$t_{g\to s}$
0.5	3.67±1.65	12.71±3.07	40.17±2.31	33.53±2.56	5.31±1.54	10.60±1.68
5.0	5.63±0.51	7.81±0.85	41.91±0.22	39.91±0.68	5.51±1.16	7.15±0.72

Removed 20% of specialists with lowest abundance and 20% of generalists with low entropy
Statistics derived from a 1,000-step Markov Chain Monte Carlo simulation

α	λ_s	μ_s	λ_g	μ_g	$t_{s\to g}$	$t_{g\to s}$
0.5	5.13±2.32	10.42±2.97	39.59±3.81	35.23±5.10	5.00±2.08	8.55±2.35
5.0	1.42±0.87	21.49±1.49	36.71±0.19	23.80±0.21	2.65±0.34	14.65±0.31

Supplementary Figure 5 | Evaluation of the impact of potential misclassification of generalists and specialists. (**a**) An illustration of a scenario where rare species may be misclassified as specialists. The dashed bars indicate hypothetical undetected abundance due to detection limit. (**b**) An illustration of a scenario where multi-environment species that are dominant in only one or a few habitats may resemble more of a specialist than a generalist. (**c**) Re-estimation of evolutionary characteristics after 5%, 10%, or 20% of classified specialists and generalists were removed. Specialists were ranked by their relative abundance (i.e. the ratio between the number of 16S rRNA fragments mapped to each of them over the total number of fragment in their respective environment, Methods). As illustrated in (**a**), species that are most susceptible to misclassification as specialist due to rarity would be those with the lowest abundance. Generalists, on the other hand, were ranked by Shannon entropy (i.e. Shannon's diversity index) calculated from the distribution of their abundance across environment clusters. As illustrated in (**b**), multi-environment species with low entropy may resemble specialists but were misclassified as generalists. Although the estimated speciation and extinction rates for generalists had high deviations, the speciation rates were consistently larger than the extinction rates with mean difference of 4.37 and standard deviation of 2.17.

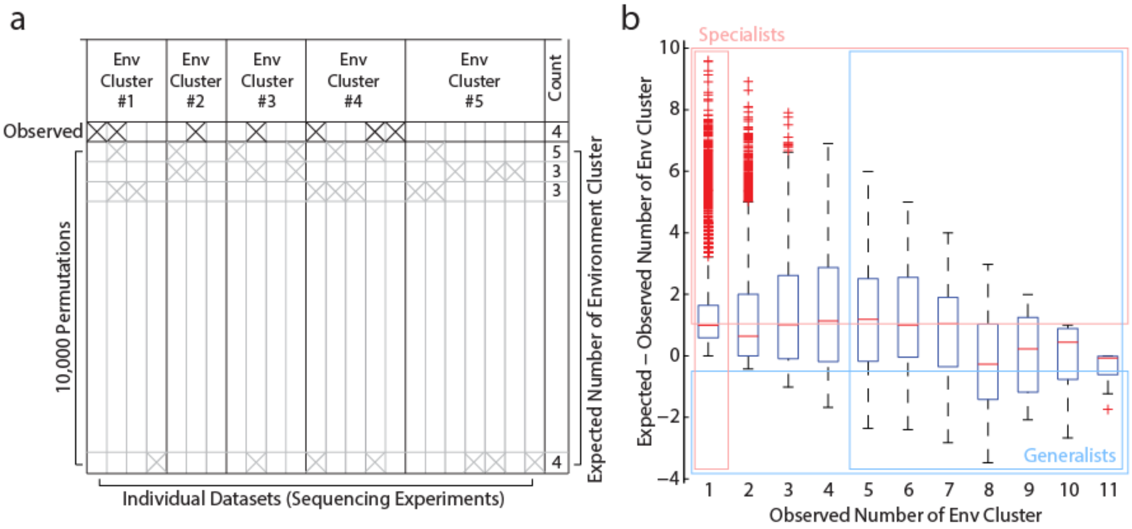

c

Using all species (statistics derived from a 1,000-step Markov Chain Monte Carlo simulation)

α	λ_s	μ_s	λ_g	μ_g	$t_{s\rightarrow g}$	$t_{g\rightarrow s}$
0.2	4.74±0.65	13.01±1.42	71.23±6.52	10.30±9.73	9.45±1.57	117.75±12.32
0.5	4.86±0.64	14.95±0.93	67.81±3.60	3.34±2.45	8.12±1.52	115.33±12.10
1.0	5.14±0.47	14.54±0.77	62.58±3.32	2.19±1.71	6.69±0.64	103.29±5.51
2.5	4.97±0.27	15.71±0.47	68.66±1.51	5.52±3.43	9.76±0.76	120.46±1.71
5.0	5.08±0.20	15.12±0.22	65.18±1.56	0.48±0.32	7.12±0.25	110.64±1.00

Using 80% of all species (statistics derived from 100 subsamples)

α	λ_s	μ_s	λ_g	μ_g	$t_{s\rightarrow g}$	$t_{g\rightarrow s}$
0.5	3.98±0.38	13.57±0.38	61.72±2.66	0.00±0.00	8.34±0.86	114.38±7.80
5.0	4.15±0.46	11.97±2.21	69.15±22.27	20.90±36.30	8.77±3.25	100.40±12.76

Supplementary Figure 6 | Alternative classification of generalists and specialists. (**a**) Illustration of the permutation-based method for defining generalists and specialists. For each species, its pattern of occurrence across all datasets (at the level of experiment accessions as listed in Table S1) was shuffled 10,000 times and the expected numbers of environment clusters were calculated. The difference between the observed and expected numbers of environment clusters was then used to classify species as generalists (observed number is larger) or specialists (expected number is larger). (**b**) Boxplots showing the relationship between the two classification methods. The blue boxes and red bars indicate the 25th-75th percentile region and the median, respectively. The black whiskers cover roughly 99.3% of the distributions. Thresholds on the difference between the observed and expected numbers of environment clusters were selected so that the numbers of classified specialists and generalists (dashed pink and cyan boxes) were the same as those derived from the other method (solid orange and cyan boxes). (**c**) Estimated evolutionary characteristics for generalists and specialists classified by the permutation-based method. Estimated extinction rates for generalists were consistently below 0.001 for all 100 subsamples. Although the estimated speciation and extinction rates for generalists had high deviations, the speciation rates were consistently larger than the extinction rates with mean difference of 48.25 and standard deviation of 15.09.

Supplementary Table 1 | Number of species as defined by fragment or full-length 16s rRNA clusters in each environment

**Environment**	**Number of Species (Fragment 16s rRNA)[a]**	**Number of Species (Full-Length 16s rRNA)[a]**
activated_carbon	383	152
activated_sludge	4766	1999
anaerobic_digester	872	440
ant	509	226
ant_fungus_garden	2072	725
aquatic	2263	868
beach_sand	141	62
beetle	174	50
biofilm	667	338
bioreactor	11903	3773
bioreactor_sludge	7245	2474
bovine	820	263
bovine_gut	9580	2242
chicken_gut	159	82
compost	331	129
coral	289	184
epibiont	18	6
fish	1796	782
food	1596	862
food_fermentation	469	159
fossil	264	57
freshwater	14401	4261
freshwater_sediment	1860	785
groundwater	13347	3473
gut	9510	3448
honeybee	155	24
hot_springs	1237	540
human	6832	2807
human_gut	6751	2155
human_lung	919	455
human_nasal_pharyngeal	812	243
human_oral	1850	874
human_skin	3032	1618
hydrocarbon	2345	1113
hydrothermal_vent	379	186

Supplementary Table 1 | Contintuted

Environment	Number of Species (Fragment 16s rRNA)[a]	Number of Species (Full-Length 16s rRNA)[a]
hypersaline_lake	251	82
ice	107	44
indoor	11	0
marine	12725	3397
marine_sediment	3793	1441
microbial_mat	179	8
mine_drainage	106	30
mosquito	684	403
mouse_gut	5600	1765
oil_production_facility	390	243
oral	12	6
phyllosphere	253	81
pig	1498	394
rhizosphere	9238	2532
rock	132	61
root	603	299
saltern	100	29
sediment	7063	2294
shoot	693	254
soil	65763	7631
stromatolite	243	110
symbiont	958	431
termite	103	28
termite_gut	48	8
wasp	49	15
wastewater	1266	603

[a] See Methods for details on how species are defined through clustering of fragment or full-length 16s rRNA sequences

Supplementary Table 2 | Average numbers of habitats for microbes from different phyla

**Phylum**	**Number of Species**	**Mean Habitat Count (Environment Cluster)**	**Mean Habitat Count of Non-Member**[a]	**U Test P-value**	**Classification (P-value < 0.001)**
Fusobacteria	85	3.717647059	2.099272139	1.98573E-14	Toward Generalist
Actinobacteria	2331	2.796653797	2.033090197	1.68625E-69	Toward Generalist
Proteobacteria	6696	2.401284349	1.995253601	2.12811E-48	Toward Generalist
Chlorobi	111	2.126126126	2.104719883	0.009400399	
Firmicutes	4902	2.01876785	2.125998092	0.006341945	
Fibrobacteres	31	2	2.10494674	0.466600626	
Thermomicrobia	26	2	2.104925572	0.487095722	
Cyanobacteria	495	1.95959596	2.107771382	0.081877816	
Nitrospirae	147	1.93877551	2.105805092	0.83337949	
Bacteroidetes	3498	1.913093196	2.136276211	5.36075E-07	Toward Specialist
Deferribacteres	10	1.9	2.104898206	0.668975253	
Chloroflexi	875	1.853714286	2.113993317	3.32912E-06	Toward Specialist
Spirochaetes	234	1.841880342	2.107318661	0.025155372	
Acidobacteria	1383	1.840925524	2.120390918	0.001994831	
Chlamydiae	17	1.823529412	2.105008468	0.854917619	
Verrucomicrobia	380	1.776315789	2.109924289	1.3935E-05	Toward Specialist
Aquificae	8	1.75	2.10493006	0.631164851	
Gemmatimonadetes	261	1.739463602	2.108699194	0.00500573	
Planctomycetes	1118	1.723613596	2.122800354	9.58605E-09	Toward Specialist
Elusimicrobia	32	1.6875	2.105354477	0.357929691	
Caldiserica	6	1.666666667	2.104921601	0.000428433	Toward Specialist
Synergistetes	39	1.538461538	2.105707136	0.008564971	
Lentisphaerae	106	1.528301887	2.107288842	0.000109682	Toward Specialist
Thermotogae	12	1.5	2.105108253	0.001750527	
Armatimonadetes	129	1.472868217	2.108117962	4.78004E-05	Toward Specialist

Supplementary Table 2 | Continued

Phylum	Number of Species	Mean Habitat Count (Environment Cluster)	Mean Habitat Count of Non-Members[a]	U Test P-value	Classification (P-value < 0.001)
Tenericutes	162	1.469135802	2.10899282	3.09222E-08	Toward Specialist
Dictyoglomi	1	1	2.10486016	0.094246235	

[a] Non-members refers species not belonging to that phylum
