# Supplementary material for: Generalist species drive microbial dispersion and evolution
Source: Nat Commun. 2017 Oct 27;8:1162. doi: 10.1038/s41467-017-01265-1 (PMC5660117; doi:10.1038/s41467-017-01265-1)
Supplement: Supplementary file 3 — Description of Additional Supplementary Files [file 41467_2017_1265_MOESM3_ESM.pdf]

Description of Additional Supplementary Files

File Name: Supplementary Data 1
Description: List of DDBJ sequence read archive entries analyzed.

File Name: Supplementary Data 2
Description: Environment association for full-length 16S rRNA clusters.

File Name: Supplementary Data 3
Description: List of classified generalists and specialists.

File Name: Supplementary Data 4
Description: Mapping of classified generalists and specialists to phylogenetic tree.
